# Spectroscopic Characterization of Emulsions Generated with a New Laser-Assisted Device

**DOI:** 10.3390/molecules25071729

**Published:** 2020-04-09

**Authors:** Andra Dinache, Tatiana Tozar, Adriana Smarandache, Ionut Relu Andrei, Simona Nistorescu, Viorel Nastasa, Angela Staicu, Mihail-Lucian Pascu, Mihaela Oana Romanitan

**Affiliations:** 1 National Institute for Laser, Plasma and Radiation Physics, 077125 Magurele, Ilfov, Romania; tatiana.alexandru@inflpr.ro (T.T.); adriana.smarandache@inflpr.ro (A.S.); ionut.andrei@inflpr.ro (I.R.A.); simona.stroescu@inflpr.ro (S.N.); viorel.nastasa@inflpr.ro (V.N.); angela.staicu@inflpr.ro (A.S.); mihai.pascu@inflpr.ro (M.-L.P.); 2 Extreme Light Infrastructure-Nuclear Physics ELI-NP, “Horia Hulubei” National Institute for Physics and Nuclear Engineering IFIN-HH, 077125 Bucharest-Magurele, Romania; 3 Physics Faculty, University of Bucharest, 077125 Magurele, Ilfov, Romania; 4 Department of Emergency Internal Medicine and Neurology, Karolinska Institute Stroke Research Network at Södersjukhuset, Stockholm South General Hospital, 118 83 Stockholm, Sweden; mihaela.romanitan@sll.se

**Keywords:** emulsion, FTIR spectroscopy, Raman spectroscopy, UV–Vis–NIR reflectance spectroscopy

## Abstract

This paper presents a spectroscopic study of emulsions generated with a laser-assisted device. Fourier transform infrared (FTIR), Raman and UV–Vis–NIR reflectance spectra of emulsions, recorded before and after exposure to laser radiation were used to characterize the effect of laser irradiation. The paper also presents a comparison between the calculated IR spectra and the experimental FTIR spectra of an emulsion’s components. FTIR measurements allowed the identification of absorption bands specific to each of the emulsions’ components. Moreover, it enabled the observation of destabilization of the emulsion in real-time. Raman spectroscopy allowed the observation of the modifications at a molecular level, by identifying the vibrations of the representative functional groups and the polymerization of sodium tetradecyl sulfate (STS) molecules by analyzing the evolution of the carbonyl band. UV–Vis–NIR reflectance spectra of emulsions before and after exposure to laser radiation showed that the physical characteristics of the emulsions changed during irradiation—the dimensions of the droplets decreased, leading to an emulsion with a better time stability. These results proved that the employed spectroscopy techniques were powerful tools in emulsion analysis.

## 1. Introduction

Emulsions are studied extensively due to their wide range of applications—targeted drug delivery, food and beverage, as well as cosmetics production, fuel and enhanced oil recovery, decontamination of surfaces, etc. [1,2,3,4,5,6,7,8]. For many of these applications, micro- and nano-emulsions present a greater interest than macro-emulsions. For example, emulsions with micro- and nano-size droplets are preferred in the medical and pharmaceutical fields, one of their advantages being longer shelf lives [9]. 

Methods of emulsification can be categorized as—high-energy and low-energy methods, or a combination of two methods from each category. Emulsification through high-energy methods implies a reduction of droplet size through disruptive forces generated with mechanical devices, e.g., microfluidizer, ultrasonication, and piston gap homogenizer. In contrast, low-energy methods of emulsification like phase inversion and spontaneous emulsification, use the internal energy of an emulsion to generate lower size droplets [10,11]. 

A new method of emulsification was described in [12]. A solution of sodium tetradecyl sulfate (STS) in water, 10% concentration, and oily vitamin A were mixed with a double-syringe system and then exposed to laser radiation, obtaining an emulsion with nano- and micro-droplets. The effect of laser radiation on emulsions is mainly mechanic, as the emulsions were irradiated at a wavelength where the immiscible components do not absorb. Therefore, the energy of laser radiation breaks the previously formed droplets and leads to generation of smaller droplets. 

In case of resonant interaction of laser beam with solution samples, a modification of molecules might appear and new photoproducts might be generated. These modifications were observed for several types of molecules, e.g., phenothiazines [13,14], antibiotics [15], hydantoins [16,17], ecdysteroids [18]. Additionally, resonant interaction of laser radiation with pendant microdroplets containing organic dye emulsions leads to enhanced fluorescence emission in comparison with the fluorescence emitted by the microdroplets containing only the laser dye in water [19]. 

At the same time, the use of emulsions requires a proper characterization of their content as well as their evolution in time. To investigate the microfluidic stability of emulsions, we analyzed the behavior of the droplets from the two phases of emulsions (oily vitamin A and STS in water). This is described by the coalescence of droplets, which leads to the separation of the immiscible liquids mixed and distributed within them [20,21]. In our case, the reduced dimensions of the droplets after irradiation, together with surfactant action of the STS molecules, led to a better stability of the irradiated emulsion when compared to one unexposed to laser radiation. Another property is the emulsion structure in terms of dimensions of constituent drops [12]. The molecular content of emulsions is another parameter and it is important to know this, since applications of emulsions depend basically on their molecular interactions with different targets. 

UV–Vis absorption spectroscopy was proven to be a powerful tool in analysis of emulsions. Absorption and scattering spectra of emulsions might provide information about the chemical composition of emulsion constituents, particle size distribution, and particle shapes [22]. 

Another spectroscopy technique employed in the study of emulsions is Fourier transform infrared (FTIR), especially, attenuated total reflection FTIR spectroscopy (FTIR–ATR). For example, it was evidenced through FTIR that, when integrated with water-in-oil (W/O) emulsions, a small percentage of the secondary structures of proteins was modified [23]. The same technique was employed to study the states of water in microemulsions [24] and to identify the signals from bulk water and from the water in the interfacial layer of reverse micelles [25]. FTIR–ATR analyzed the water structure near the surface of nanoparticles in W/O microemulsions [26]. Other FTIR–ATR studies showed that the emulsifier interacts with water molecules in the interfacial layer of W/O emulsions, weakening the hydrogen bonding network and preventing coalescence of water droplets [27]. Moreover, the effect of emulsifiers concentration on the stabilization of emulsions was studied, using the –OH stretching vibration band as a sensor for the molecular interactions that lead to the stabilization of W/O emulsion [28]. 

Complementary to previous spectroscopy techniques, Raman spectroscopy was employed in emulsions characterization. Studies showed that the gluten protein was modified after adding varying concentrations of an emulsifier [29]. Raman spectroscopy was proven to be better than calorimetry at determining free monomer concentrations, while monitoring emulsion polymerization reactions [30]. 

In this paper, we present the spectroscopic characterization of emulsions generated with a laser-assisted device and the comparison of UV–Vis reflectance, FTIR–ATR, and Raman spectra of the unirradiated and irradiated emulsions. A comparison between the theoretical IR spectra and the experimental FTIR spectra of the emulsion’s components is also presented. 

## 2. Results and Discussions 

### 2.1. FTIR Spectroscopy 

**Figure 1:**
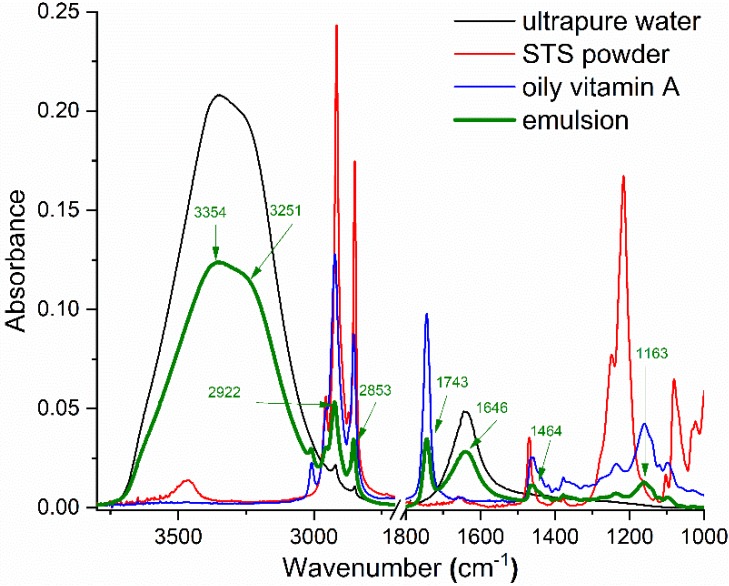
FTIR–ATR spectra of emulsion and its components (ultrapure water, sodium tetradecyl sulfate (STS) powder, and oily vitamin A).

FTIR–ATR spectra of the emulsion and each of its components (ultrapure water, STS powder, and oily vitamin A) are presented in Figure 1.

Two large bands with peaks at 3354 cm^−1^ and 1646 cm^−1^ characterized the IR spectrum of ultrapure water. The main peaks of the STS powder observed in the IR spectral range were: 2955 cm^−1^, 2916 cm^−1^, 2849 cm^−1^, 1468 cm^−1^, 1247 cm^−1^, 1215 cm^−1^, and 1080 cm^−1^. FTIR–ATR spectrum of oily vitamin A evidenced absorption bands with maxima at 3009 cm^−1^, 2953 cm^−1^, 2922 cm^−1^, 2853 cm^−1^, 1743 cm^−1^, 1464 cm^−1^, 1377 cm^−1^, 1232 cm^−1^, 1163 cm^−1^, and 1097 cm^−1^.

For oily vitamin A and STS 10% emulsion, 1:1 ratio, one might observe that the IR spectrum mainly showed the contribution of ultrapure water and vitamin A characteristic vibrational bands. Nevertheless, even if the STS concentration was low, it could influence the absorption band originating from the O–H stretching vibrations. STS decreased the chance of coalescence of water droplets by reducing the strength of the hydrogen bonding network [27]. 

IR domains of interest in studying these emulsions were 3600–3100 cm^−1^ (from the O–H vibrations), 3000–2800 cm^−1^ (C–H stretching vibrations give rise to absorption bands in this range), and 1800–1600 cm^−1^ (C=O vibrations). In the range of 3600–3100 cm^−1^, the band of relevance was the large absorption band with peak at 3354 cm^−1^, attributed to the O–H stretching vibrations from water molecules. Symmetrical and asymmetrical stretching vibrations of methylene (–CH_2_) from the aliphatic chain gave rise to absorption bands with peaks at 2922 cm^−1^ and 2853 cm^−1^. The band with the peak at 1743 cm^−1^ was due to the carbonyl bond vibrations from the oily vitamin A solution. Additionally, assigned to water molecules, the absorption band at 1646 cm^−1^ was generated by the bending vibrations of H–O–H.

**Figure 2:**
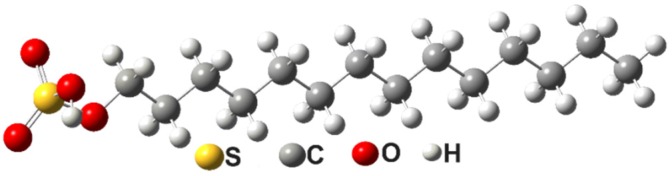
Optimized chemical structure of STS.

**Figure 3:**
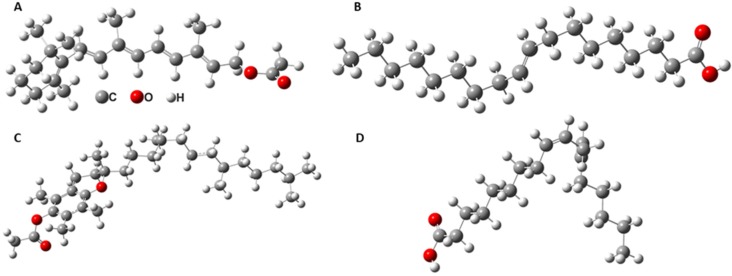
Major components of an optimized chemical structure of oily vitamin A: (**A**) vitamin A; (**B**) oleic acid; (**C**) linoleic acid; and (**D**) α-tocopherol acetate.

FTIR–ATR spectra of STS, vitamin A, α-tocopherol acetate, linoleic acid, and oleic acid (last four were considered to be the main constituents of oily vitamin A [31]) were recorded and compared with vibrational spectra calculated with the Gaussian09 software and using the DFT method. Prior to simulating the IR spectra, it was necessary to optimize the molecules’ structure. Figure 2 and Figure 3 show the optimized chemical structures of STS and the main constituents of oily vitamin A, respectively. 

The calculated wavenumbers are usually overestimated and should be corrected through the scaling functions [32,33]. The theoretical frequencies for the STS molecules, calculated as described in [34], are given in Table 1, in comparison with the experimental values. 

IR spectrum of STS molecules evidenced symmetrical and asymmetrical stretching vibrations of C–H bonds from the aliphatic chain, which were responsible for the 3015–2760 cm^−1^ bands. The bands observed between 1300–1100 cm^−1^ were due to CH_2_ out-of-plane bending from the aliphatic chain. 

Further, our study focused on identifying the IR frequencies of vitamin A (the active medical compound in oily vitamin A) in the IR spectrum of oily vitamin A. The experimental IR frequencies of oily vitamin A and the theoretical IR frequencies of vitamin A are presented in Table 2. FTIR spectrum of oily vitamin A presented absorption bands in the range 3000–2800 cm^−1^, due to the symmetrical and asymmetrical stretching vibrations of the C–H bonds from methyl groups. Stretching vibrations of the carbonyl bond gave rise to the absorption band at 1743 cm^−1^ and the stretching vibrations of the C–O bonds were responsible for the appearance of the peak at 1097 cm^−1^. The bands observed at 1464 cm^−1^ and 1377 cm^−1^ were due to the out-of-plane bending vibrations (wagging) of the C–H bonds from the CH_3_ groups, while the one at 1163 cm^−1^ was given by the C–H twisting vibrations of the CH_3_ groups. A comparison between the experimental frequencies and the calculated ones is presented in Table 2. The relative standard deviation of these values was 0.36%, suggesting a better approximation of the experimental spectrum than in the case of the STS molecules.

Moreover, in the IR spectra of oily vitamin A, IR frequencies that correspond to the main constituents of refined sunflower oil, oleic, and linoleic acids, were also observed. At 3009 cm^−1^, in the IR spectrum of oily vitamin A, C–H asymmetrical stretching from the CH_2_ groups of the oleic acid (2979 cm^−1^–theoretical frequency) and linoleic acid (3009 cm^−1^) was also represented. The band observed at 1464 cm^−1^ was also due to the C–H asymmetric deformation vibrations from the CH_2_ groups found in both oleic (1465 cm^−1^) and linoleic (1462 cm^−1^) acids. Additionally, the stretching vibrations of the C–C bonds of chains were observed in the experimental spectrum of oily vitamin A in the 1045–890 cm^−1^ range, whereas the theoretical spectrum of oleic acid were observed between 1047 cm^−1^ and 898 cm^−1^ and that of linoleic acid was observed between 1033 cm^−1^ and 908 cm^−1^.

We did not identify α-tocopherol acetate vibrational features in the IR spectrum of oily vitamin A, mainly because this was probably used in a small amount for its antioxidant properties.

**Figure 4:**
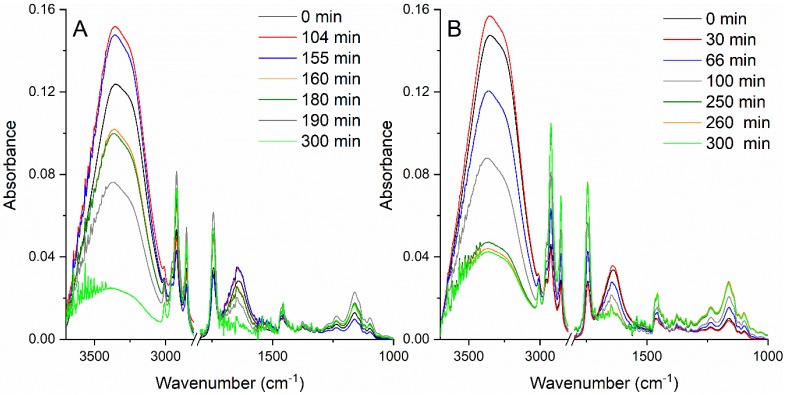
FTIR–ATR spectra of the unirradiated emulsion (**A**) and 1 h irradiated emulsion (**B**) recorded during 300 min.

FTIR–ATR spectra of emulsions were recorded during 300 min, with the purpose of studying their time stability. A comparison between the spectra of O/W emulsion (50% oily vitamin A (v/v) with the oil phase 2% vitamin A in sunflower oil and water phase 10% STS in ultrapure water) and a similar emulsion exposed to laser radiation for 1 h is presented in Figure 4. 

One might observe that the intensity of the absorption band at 3354 cm^−1^ decreased during 300 min, suggesting lesser O–H vibrations of water molecules, thus, implying a destabilization of emulsions. The same decrease in intensity was seen for the absorbance maximum at 1646 cm^−1^, which was assigned to the H–O–H bending vibrations of water molecules. These modifications in spectra occurred simultaneous to the increase of bands, with peaks at 2922 cm^−1^ and 2853 cm^−1^. The absorbance increases were related to the increment of the number of –CH_2_ vibrations, as a result of a reduction in the interactions between the STS and vitamin A molecules within the emulsion, with time evolution. A similar increase was observed for the absorption band at 1741 cm^−1^, as a result of an increase in the stretching vibrations of C=O bonds. All modifications observed over 300 min were due to a better adsorption of vitamin A at the surface of the ZnSe crystal, suggesting a continuous separation of the two phases of the emulsions. This also implied that, during the destabilization of the emulsions, part of the water droplets evaporated during the experiment (300 min), leading to a decrease of the absorption maxima originating from the water molecules and an increase of the oily vitamin A peaks.

**Figure 5:**
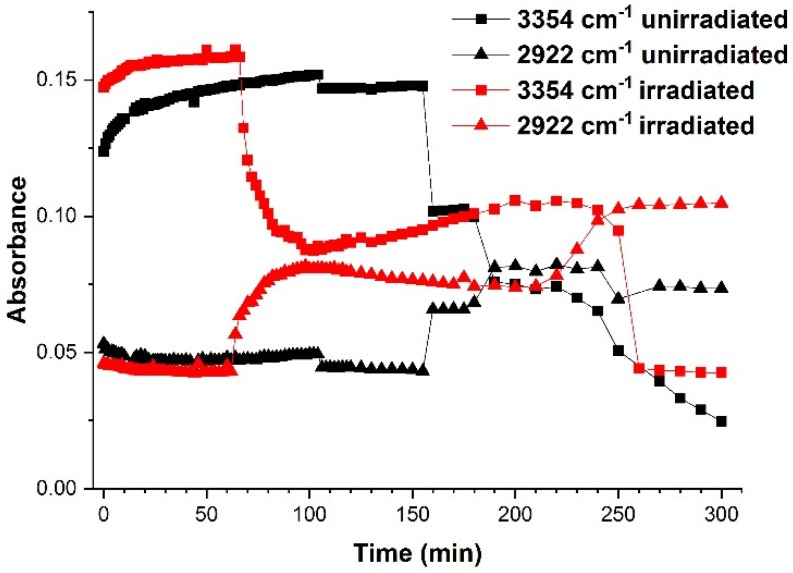
Time evolution of absorbance peaks at the 3354 cm^−1^ representative for the STS water solution and the 2922 cm^−1^ representative for oily vitamin A. Comparison between destabilization of the unirradiated emulsion (black) and the destabilization specific to the emulsion irradiated for 1 h (red).

Thus, considering the intensity modifications of the absorption bands, the time stability of the emulsions might be investigated by analyzing their FTIR–ATR spectra. Figure 5 shows the evolution of the maxima at 3354 cm^−1^ and 2922 cm^−1^, for both unirradiated and irradiated emulsions. Each absorption band was representative of one of the immiscible components of the emulsion—3354 cm^−1^ for STS solution in water and 2922 cm^−1^ for oily vitamin A. For the unirradiated emulsion, the absorbance at 3354 cm^−1^ slightly increased during 155 min, and at the same time a slow decrease was observed for the absorbance at 2922 cm^−1^. After 155 min, a steep decrease of the absorbance was observed for the 3354 cm^−1^ maximum, simultaneous to a boost of the 2922 cm^−1^ peak. The steep changes observed might be associated with the formation of a larger oil droplet, through creaming at the surface of the ATR crystal. Even if at the beginning of the measurements the absorbance at 3354 cm^−1^ increased slowly, suggesting a migration of the STS in water droplets towards the ATR crystal and a migration of oily vitamin A droplets to the upper part of the emulsion during the destabilization processes, the peak at 3354 cm^−1^ decreased by the end of the experiment. Considering the differences of the absorbance values, approximately 2/3 of the quantity of STS in water solution evaporated throughout the duration of the measurements. A similar behavior of the peaks was observed for the emulsion exposed to laser radiation for 1 h, the difference consisting in the observed time of steep decrease for the peak attributed to water vibrations, and an increase of the peak due to vitamin A vibrations. For the irradiated emulsion, these modifications were observed after 66 min. After a steep decrease of the 3354 cm^−1^ peak, and the maximum increase of 2922 cm^−1^, the curves, shown in Figure 5, continued their initial trend until similar steep modifications appeared. 

This behavior of the FTIR spectra is a real-time indication of the destabilization of emulsions. Taking into consideration that the dimensions of the droplets at the beginning of the FTIR measurements were around 270 nm for the unirradiated emulsion, and around 142 nm for the irradiated one [12], the modifications of the peak absorbance recorded in the first spectra (Figure 5) might be associated with Ostwald ripening and coalescence of the small droplets, which are processes involved in the destabilization of emulsions. First, Ostwald ripening causes nanosize droplets to form larger size droplets, due to mass transfer from smaller to larger droplets. After this, the microsize droplets merge during coalescence, leading to the appearance of bigger droplets. Once Ostwald ripening and coalescence increase the dimensions of the droplets, creaming becomes the main instability mechanism for W/O emulsion 50% (*v*/*v*) oily vitamin A. Considering that the dimensions of the droplets during the creaming process are larger than the initial ones, their migration at the surface of the ATR crystal is responsible for more significant modifications of the absorbance maxima. Destabilization of the emulsions end with a total separation of the two phases [1,12,35]. 

The steep modifications of the curves representing the time evolution of absorbance maxima were due to the formation of one large drop of vitamin A at the surface of the ZnSe crystal, hence, the increase of the absorbance peak at 2922 cm^−1^.

**Figure 6:**
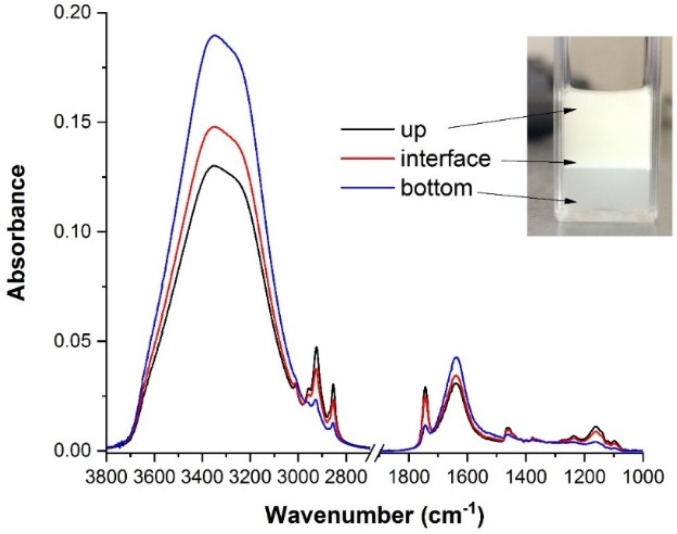
Evidence of emulsion creaming through FTIR spectra.

For a detailed observation of the phenomenon, the FTIR–ATR spectra were recorded during the creaming process from the areas of a cuvette containing the unirradiated emulsion, as shown in Figure 6. 

Creaming leads to phase separation in emulsion. At the moment of recording, the unirradiated emulsion was destabilized into two emulsions: (i) one at the bottom of the cuvette, where the dispersed phase was vitamin A, and (ii) in the upper part of the cuvette, where the dispersed phase was STS 10% solution in water. The interface area was similar to that of the initial emulsion. Recording the FTIR spectra from all three areas, evidences the creaming and, thus, the absorbances of peaks at 3354 cm^−1^ and 1646 cm^−1^ that were attributed to the vibrations of the water molecules, were higher for emulsion at the bottom of the cuvette, while, the absorbances of the peaks at 2922 cm^−1^, 2853 cm^−1^, 1743 cm^−1^, and 1163 cm^−1^ were higher for the sample in the upper area. These peaks were attributed to the vibrations of the oily vitamin A phase. Even though visual inspection of the samples could offer information about emulsions destabilization, we used FTIR–ATR analysis to follow the process more precisely and to quantify this in time.

### 2.2. Raman Spectroscopy 

One of the components of the emulsions generated by the developed laser-assisted system was STS. This is an anionic surfactant that polymerizes by emulsification with oils (in our case vitamin A) and forms the so-called latex products [36]. This process could be evidenced from a comparison of the Raman spectra registered for the emulsion as a whole and separately for its components. The modifications at a molecular level could be identified through the representative functional groups and the corresponding vibrations.

**Figure 7:**
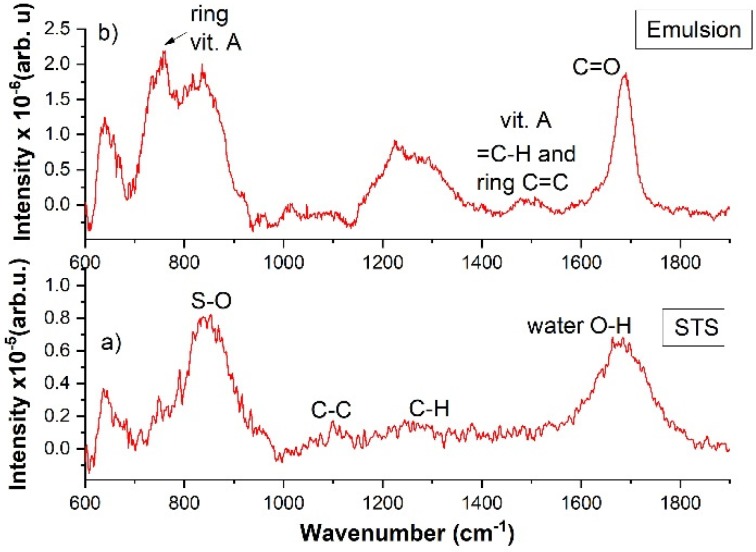
Raman spectra excited with pulsed laser beam at 266 nm, 40 mJ/per pulse for (**a**) 10% STS in water solution, (**b**) O/W emulsion (50% oily vitamin A (*v*/*v*) with the oil phase 2% vitamin A in sunflower oil, and the water phase 10% STS in ultrapure water).

Figure 7a shows the Raman spectrum for a 10% STS solution in water for the spectral range 600–1800 cm^−1^. The spectra were averaged on 2000 laser pulses. The specific vibration bands for STS could be observed: S–O symmetric stretch at 840 cm^−1^, C–C symmetric and asymmetric stretch for the aliphatic chain at 1100 cm^−1^, bending vibration of C–H from CH_2_ in the range of 1220 cm^−1^–1300 cm^−1^, and the water O–H band at 1678 cm^−1^. 

Due to emulsification with oily vitamin A, in the Raman spectrum of the emulsion (Figure 7b) one could identify the IR spectral features of the vitamin and the modification of the STS bands. The characteristic bands of vitamin A were the aromatic ring vibration at 761 cm^−1^, =C–H, and the C=C vibrations for the aromatic ring. Following the polymerization of STS, the C=O band of the carbonyl group that was formed by monomers coupling, showed up in the emulsion spectrum. In addition, an increase in the intensity for the C–H bending vibration bands of CH_2_ from the aliphatic chain could be observed at 1224 cm^−1^ and 1289 cm^−1^.

The most stable emulsion samples were the O/W emulsions (50% oily vitamin A (*v*/*v*) with the oil phase 2% vitamin A in sunflower oil and the water phase 10% STS in ultrapure water). These were analyzed by Raman spectroscopy at excitation with 532 nm pulsed laser beam. Sequences of 10 spectra averaged on 3000 laser pulses were registered during 50 min irradiation with a beam of 35 mJ energy per pulse.

**Figure 8:**
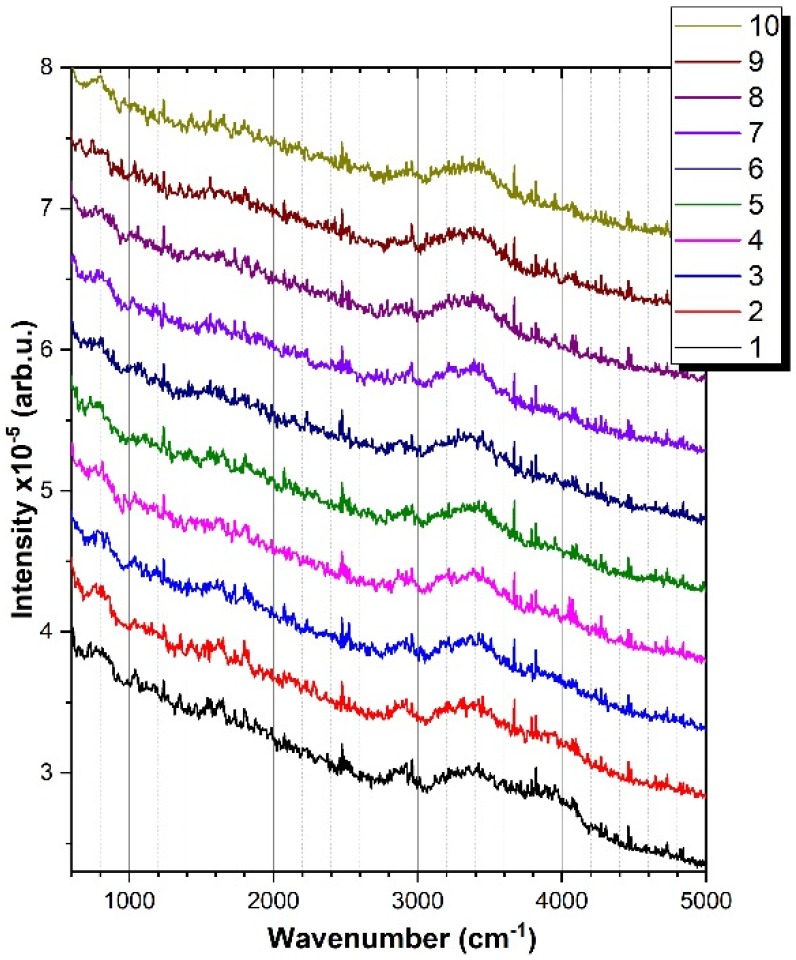
Raman spectra for an O/W emulsion (50% oily vitamin A (*v*/*v*) with the oil phase 2% vitamin A in sunflower oil and the water phase 10% STS in ultrapure water), recorded during 50 min of laser irradiation (each spectrum was averaged on 3000 laser pulses/300 s).

This spectra suite is shown in Figure 8 for the spectral range of 800–5000 cm^−1^.

During irradiation, the Raman features for vitamin A, STS, and water showed modifications that could be an evidence of structural and morphological changes of emulsion. At 3400 cm^−1^, the O–H stretching vibration was present and remained constant during irradiation. Instead, the large vibration band located at 2887 cm^−1^ and corresponding to the C–H stretching from the aliphatic chain showed a decrease in intensity during laser irradiation. This could be due to the reduction of the size of emulsion droplets that contained STS or vitamin A. The polymerization of the STS molecules could be tracked by observing the evolution of the carbonyl C=O band at 1800 cm^−1^. Additionally, unsaturated fatty acids, such as linoleic and oleic acids, which are present in the composition of sunflower oil and contain the C=O carbonyl functional group in its chemical formulation, could polymerize through emulsification. The modification of this band could be attributed to a cross-linking polymerization of STS or oil components and to a fragmentation of oil droplets with laser action on emulsion.

### 2.3. UV–Vis–NIR Reflectance Spectroscopy

The emulsion samples showed strong light scattering and, consequently, the UV–Vis–NIR transmission spectra did not exhibit conclusive results. For this reason, the investigation of their optical properties in this domain was directed to reflectance spectra analysis.

**Figure 9:**
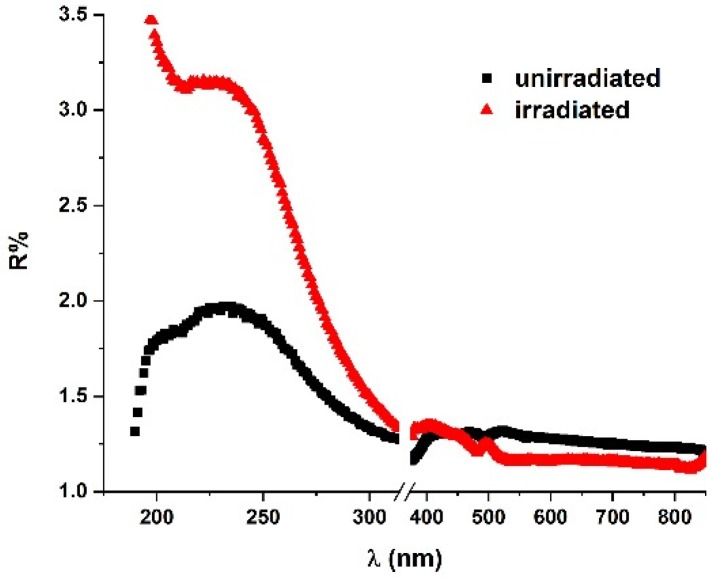
UV–Vis–NIR reflectance spectra of W/O emulsion 50% (*v*/*v*) oily vitamin A, before and after laser radiation exposure (λ = 532 nm, E = 35 mJ, t = 1 h).

Figure 9 presents the UV–Vis–NIR reflectance spectra of O/W emulsions (50% oily vitamin A (*v*/*v*) with the oil phase 2% vitamin A in sunflower oil and the water phase 10% STS in ultrapure water), before and after exposure to laser radiation. The analysis of these spectra allowed the identification of a maximum at 234 nm for the unirradiated emulsion, while after the laser beam exposure, a blue shift of this peak (Δʎ = 10 nm, hypsochromic effect) was noted, along with the rise of its intensity (hyperchromic effect). The 234 nm feature could be related to the absorption band of vitamin A. The intensity increase of this band after laser exposure suggests the shrinking of oily droplets and an enlargement of their apparent reflection surface. This was in agreement with the decrease of the oily droplets reported in [12]. The hydrodynamic diameter of the droplets decreased from 269.5 nm to 141.8 nm after irradiation. More so, decrease and uniformization of the droplets increased the stability of emulsions [37], which could be inferred from the zeta potential and surface tension measurements performed on the emulsions, before and after exposure to laser radiation [12]. 

These aspects, associated with other spectroscopic measurements and previously reported analysis [12] through optical microscopy, dynamic light scattering, surface tension analysis, etc., led to the following conclusions (1) the physical characteristics of the emulsion changed subsequent to laser radiation exposure concerning both a decrease of the microdroplets dimensions and variations of the interface properties; and (2) laser exposure improved the emulsion stability. 

## 3. Materials and Methods

### 3.1. Materials

Analyzed emulsions contained STS solutions in ultrapure deionized water and oily vitamin A.

STS is an anionic detergent used in sclerotherapy of legs varicose veins [38] and it was successful in treating venous malformations of the head and the neck [39]. The concentration of the STS (95% purity, purchased from Sigma) solutions in water was 100 mg/mL (10%), below the critical micelle concentration (CMC) [40]. During experiments, STS solutions were kept in dark, at room temperature. 

The ultrapure de-ionized water was delivered via a sterile filter. Its bacterial content was <1CFU/mL and the particle content was <1 (TKA Smart2Pure UV), at a resistivity of 18.2 MΩ × cm and 0.055 μS/cm conductivity, at 25 °C. 

Vitamin A plays an important role in preventing a series of eye diseases and moreover, it was observed in some developing countries that vitamin A supplementation reduces infant mortality [41]. The concentration of vitamin A in oil (Biofarm, Romania) was 20 mg/mL (2%) and had refined sunflower oil and α-tocopherol acetate as excipients. The major constituents of sunflower oil are linoleic acid (52.38%) and oleic acid (34.5%) [31]. In this context, we considered α-tocopherol acetate, linoleic acid, oleic acid, and vitamin A to be the major constituents of oily vitamin A. Oily vitamin A did not contain any surface-active components. To attest this, surface tension analysis was performed by the pendant drop technique, prior to all spectral analyses (data not shown). The solutions were kept in dark, at 4 °C, but were acclimated to room temperature before the experiments.

### 3.2. Experimental System

**Figure 10:**
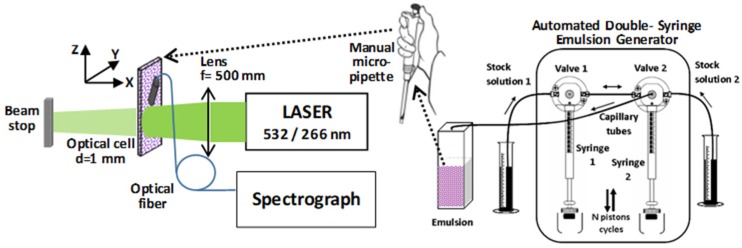
Scheme of the laser-assisted device that consist of an automated double-syringe emulsion generator unit and a laser emulsion processing unit.

The laser-assisted device was described in detail in [12]. This consisted of an automated double-syringe emulsion generator unit and a laser emulsion processing unit (Figure 10). The automated generator unit was represented by a double syringe diluter–dispenser commercial system (Hamilton ML 600 System, USA) that was computer controlled. A control software was specifically developed to realize a sequence that included fill–mix–dispense steps with controlled parameters, such as, solutions’ loading speed from stock solutions 1 and 2, number N of mixing cycles between syringes, mixing speed, and emulsion expelling speed [12]. The resulted emulsion was then transferred via a manual micropipette to a spectrophotometric cell of 1 mm optical path and was then subjected to laser processing. The emulsion was not mixed during irradiation. The laser emulsion processing unit was formed by a Nd:YAG laser (Surelite II, Continuum, Excel Technology, Santa Clara, CA, USA) and an irradiation optical system. The pulsed beam, 10 pps frequency, pulse duration (FTWHM) 6 ns, wavelength λ = 532 or 266 nm, average energy of 35 mJ was processed with a lens of a focal length of 500 mm, so that the beam covered the cell width. The same irradiation area (of 8 mm diameter) of the cell was maintained through the entire period of irradiation. For Raman spectroscopy, the same laser excitation source (second/fourth harmonic of the Nd:YAG laser) was used as for the emulsion irradiation. The Raman scattered signals were collected with an optical fiber (1 mm core diameter) placed at an angle of 45° in the XZ plane and was sent for detection and analysis to a spectrograph—ICCD system (Acton Research/Princeton Instruments, Spectra Pro, model SP-2750, ICCD–PIMAX 1024 RB).

### 3.3. Methods

Emulsions were generated as described in [12], mixing the STS solution in ultrapure deionized water with oily vitamin A, without any additional surfactant. All spectroscopic measurements were performed on O/W emulsions (50% oily vitamin A (*v*/*v*) with the oil phase 2% vitamin A in sunflower oil and the water phase 10% STS in ultrapure water) generated in 800 mixing cycles, and similar emulsions were irradiated for 1 h. The sample from the irradiated emulsion to be analyzed was collected with a micropipette, from the area exposed to the laser beam.

IR spectra of the emulsions were recorded using the attenuated total reflection (ATR) module of the FT-IR Nicolet™ iS™50 spectrometer, as a mean of 16 spectra, with a resolution of 4 cm^−1^. A 50-µL volume emulsion was placed into a ring sample holder that ensured the same contact surface for all samples on the ZnSe crystal, and 100 spectra were acquired at increasing time intervals, up to 300 min. The ZnSe crystal had the following characteristics—one internal reflection at an incidence angle of 42°, 1.5 mm diameter, 2.4 refractive index, and a depth of penetration of 2.03 µm, at 1000 cm^−1^.

Spectra acquired this way were compared with theoretical IR spectra, calculated with Gaussian software suite [42]. In order to obtain the calculated IR spectra, first the molecular structures were drawn in GaussView 5.0. and then the molecules were optimized from the geometric and structural points of view with Gaussian 09. Opt+Freq (Optimization and Frequency); job type was selected to perform the calculations. Vibrational frequencies of the molecules were calculated using a computational quantum mechanical modeling method—density functional theory (DFT) with the B3LYP (Becke, 3-parameter, Lee-Yang-Parr) method and 6-311G(d,p) basis set. This method successfully utilized the functionals of the electron density to investigate the electronic structures of molecules [43,44].

The experimental set-up for Raman measurements is described in detail elsewhere [45,46,47].

Raman spectra were collected for the 600–1800 cm^−1^ and 800–5000 cm^−1^ spectral ranges, with a resolution of 0.3 nm, and was averaged on 2000 or 3000 signals.

The reflectance of the emulsions was analyzed using the specular reflection technique, immediately after preparation, as well as following exposure to laser beam (ʎ = 532 nm; E = 35 mJ), for 1h. A fixed angle (at 6°) reflectance accessory was used, which was mounted in the sample compartment of the Lambda 950 UV–Vis–NIR absorption spectrophotometer (Perkin Elmer, USA). The spectra were recorded between 180 nm and 900 nm.

## 4. Conclusions

The spectroscopy techniques proved to be powerful tools in the analysis of emulsions. FTIR analyses of emulsions allow the identification of absorption bands specific to each of the immiscible components, further allowing a real-time evaluation of the emulsion’s destabilization. The calculated IR spectra were in good agreement with the experimental FTIR spectra of the emulsion’s components. FTIR–ATR spectra allowed the identification of the action of STS emulsifier on water molecules, even if its concentration was too low to be measured. Its influence on water molecules might be observed by the FTIR–ATR in the absorption band, due to the O–H stretching vibrations. Raman spectroscopy enabled the characterization of the modifications at the molecular level, through identification of the corresponding vibrations from the functional groups of emulsion components. This technique also allowed the observation of the polymerization of STS molecules by analyzing the modifications of the C=O band at 1800 cm^−1^. The difference between the UV–Vis–NIR reflectance spectra of the unirradiated and irradiated emulsions (correlated with previous reported measurements) showed that the physical characteristics of the emulsions changed during exposure to laser radiation. The increase in the reflectance signal suggests that the dimensions of the droplets decreased, leading to an emulsion with better time stability.

All these techniques showed promising results in analysis of emulsions at the molecular level, as well as characterizing their droplets dimensions and time stability.

## Figures and Tables

**Table 1:** Experimental and calculated vibration frequencies for STS.

Calculated ν (cm^−1^)	Experimental ν (cm^−1^)	Assigned Vibrations
2960	2955	C–H asymmetrical stretching from CH_3_ from aliphatic chain
2939	2916	C–H asymmetrical stretching from CH_2_ from aliphatic chain
2897	2849	C–H symmetrical stretching from CH_2_ from aliphatic chain
1393	1247	CH_2_ out of plane bending from aliphatic chain
1373	1215	CH_2_ out of plane bending from aliphatic chain

The relative standard deviation between calculated and experimental frequencies was <6.1%.

**Table 2:** Experimental vibrational frequencies for oily vitamin A and the calculated vibration frequencies for vitamin A.

Calculated ν (cm^−1^)	Experimental ν (cm^−1^)	Assigned Vibrations
3008	3009	C–H asymmetrical stretching from CH_3_ groups
2932	2953	C–H asymmetrical stretching from CH_3_ groups
2910	2922	C–H symmetrical stretching from CH_3_ groups
2856	2853	C–H symmetrical stretching from CH_3_ groups
1747	1743	C=O stretching
1466	1464	C–H out of plane bending (wagging) from CH_3_ groups
1377	1377	C–H out of plane bending (wagging) from CH_3_ groups
1163	1163	C–H out of plane bending (twisting) from CH_3_ groups
1025	1097	C–O stretching
